# Band-Gap Tunability in Anharmonic Perovskite-Like Semiconductors Driven by Polar Electron–Phonon Coupling

**DOI:** 10.1021/jacs.5c11968

**Published:** 2025-09-30

**Authors:** Pol Benítez, Ruoshi Jiang, Siyu Chen, Cibrán López, Josep-Lluís Tamarit, Edgardo Saucedo, Bartomeu Monserrat, Claudio Cazorla

**Affiliations:** † Department of Physics, Universitat Politècnica de Catalunya, Barcelona 08034, Spain; ‡ Research Center in Multiscale Science and Engineering, Universitat Politècnica de Catalunya, Barcelona 08019, Spain; § Department of Materials Science and Metallurgy, 2152 University of Cambridge, Cambridge CB3 0FS, U.K.; ∥ Department of Electronic Engineering, Universitat Politècnica de Catalunya, Barcelona 08034, Spain

## Abstract

The ability to finely tune optoelectronic properties in semiconductors is crucial for the development of advanced technologies, ranging from photodetectors to photovoltaics. In this work, we propose a novel strategy to achieve such tunability by utilizing electric fields to excite low-energy polar optical phonon modes, which strongly couple to electronic states in anharmonic semiconductors. We conducted a high-throughput screening of over 10,000 materials, focusing on centrosymmetric compounds with imaginary polar phonon modes and suitable band gaps, and identified 310 promising candidates with potential for enhanced optoelectronic tunability. From this set, three perovskite-like compounds—Ag_3_SBr, BaTiO_3_, and PbHfO_3_—were selected for in-depth investigation based on their contrasting band gap behavior with temperature. Using first-principles calculations, ab initio molecular dynamics simulations, tight-binding models, and anharmonic Fröhlich theory, we analyzed the underlying physical mechanisms. Our results show that polar phonon distortions can induce substantial band gap modulations at ambient conditions, including reductions of up to 70% in Ag_3_SBr and increases of nearly 23% in BaTiO_3_, relative to values calculated at zero temperature, while PbHfO_3_ exhibits minimal change. These contrasting responses arise from distinct electron–phonon coupling mechanisms and orbital hybridization at the band edges. This work establishes key design principles for harnessing polar lattice dynamics to engineer tunable optoelectronic properties, paving the way for adaptive technologies such as wavelength-selective optical devices and solar absorbers.

## Graphical abstract

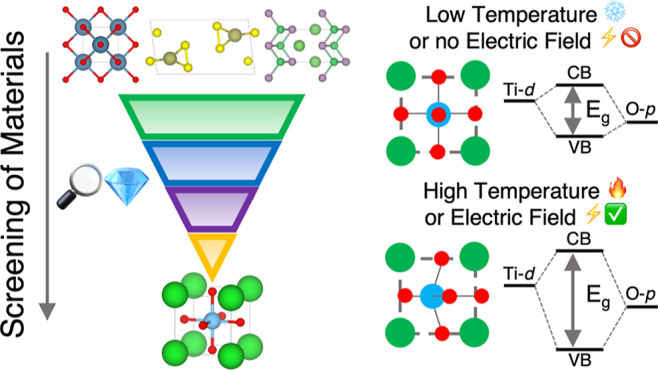

## Introduction

Semiconductors are foundational to modern technologies and play a critical role in a wide range of applications, including optoelectronics, computing, information storage, and energy harvesting. A defining feature of these materials is the electronic band gap, namely, the energy difference between the valence band maximum (VBM) and the conduction band minimum (CBM), which typically spans a few eV. Achieving significant externally controlled band gap variations in semiconductors is crucial for enabling tunable electronic and optoelectronic devices, such as transistors, sensors, and photodetectors.

The band gap of semiconductors is known to vary with temperature,^1,2^ primarily due to electron–phonon interactions^3^ and thermal expansion of the crystal lattice.^4^ While these variations are typically modest, on the order of a few meV,^5–7^ certain materials exhibit much larger shifts, reaching several hundred meV at room temperature.^8,9^ For most compounds, the band gap decreases with increasing temperature, a trend well described by the empirical Varshni relation.^10^ However, anomalous cases also exist,^11^ where the band gap increases with temperature. This behavior can manifest in two forms: a nonmonotonic dependence, in which the band gap initially increases and then decreases, and a monotonic increase over the entire temperature range. Representative examples include the chalcopyrite ZnSnAs_2_ ^12^ (nonmonotonic) and the copper halide CuCl^13^ (monotonic). It is important to clarify that the term anomalous is commonly used to describe the increase of the band gap with temperature, which contrasts with the trend predicted by the empirical Varshni relation. However, this behavior can be theoretically understood within the framework of harmonic Allen–Heine–Cardona theory.^12–14^

Although temperature can influence the optoelectronic properties of semiconductors through mechanisms such as electron–phonon interactions and thermal expansion, it is inherently limited as a tool for dynamic control. In practical applications, such as tunable photodetectors, adaptive photovoltaics, or reconfigurable optoelectronic devices, one requires fast, reversible, and spatially controlled modulation of material properties. Temperature changes are typically slow, energetically inefficient, and lack spatial precision. A more practical and versatile approach would involve applying external stimuli, such as electric fields, that can dynamically and selectively manipulate the electronic structure. In this context, identifying mechanisms by which external fields can induce band gap modifications, particularly through their interaction with the lattice, is of critical importance for the development of next-generation functional materials.

On a more fundamental level, despite the availability of extensive empirical data on temperature-dependent band gap shifts, predictive frameworks for anticipating such behavior in unexplored materials remain limited. Simple and chemically intuitive descriptors, such as elemental composition, bonding characteristics, or crystal symmetry, often fail to reliably predict whether a material will exhibit significant, negligible, or anomalous band gap variations with temperature. In this regard, anharmonic lattice dynamics, characterized by low-energy and large-amplitude phonons, may strongly impact electron–phonon interactions, either by enhancing or reducing harmonic thermal effects on the band gap.^15–17^ Nevertheless, the microscopic mechanisms linking anharmonicity to electronic structure renormalization remain poorly understood and are seldom captured by conventional theoretical models. Few recent studies have begun to explore this connection in specific materials, such as SrTiO_3_ ^18^ and CuInTe_2_,^19^ providing valuable insights into how anharmonic effects might be harnessed to design materials with tunable optoelectronic properties.

In this work, we propose a set of electronic and lattice vibrational criteria to identify materials with the potential for large band gap variations, whether driven by temperature or external electric fields. Based on these criteria, we perform a high-throughput computational screening of a large materials database comprising thousands of precomputed electronic band structures and phonon spectra, ultimately identifying over 300 promising candidates. From this set, we select three highly anharmonic perovskite-like compounds, namely, Ag_3_SBr, BaTiO_3_ and PbHfO_3_, for in-depth investigation using a combination of advanced computational methods, including first-principles density functional theory (DFT), ab initio molecular dynamics (AIMD), tight-binding models, and anharmonic Fröhlich theory.

Our analysis uncovers a set of simple, chemically intuitive mechanisms that account for the observed trends in temperature-induced band gap variations. In particular, strong electron–phonon coupling mediated by low-frequency polar phonon modes can give rise to either conventional Varshni-like behavior or anomalous temperature dependencies, depending on the specific orbital hybridizations at the band edges. These findings demonstrate the feasibility of rationally designing optoelectronic materials whose properties can be tuned through phonon-mediated interactions, potentially enhanced by external electric fields, thereby opening avenues for innovative technologies.

## Results

We begin this section by detailing the criteria used for the high-throughput screening of semiconductor materials with potentially high optoelectronic tunability, along with the rationale behind these choices. We then discuss the most promising candidates identified in our search and present a refinement of the precomputed first-principles data associated with them. Based on band gap calculations performed on frozen-phonon distorted configurations, we select three representative compounds, all exhibiting perovskite-like structures although contrasting temperature-dependent band gap behaviors, for a detailed investigation. Finally, we elucidate the underlying electron–phonon coupling and orbital hybridization mechanisms responsible for the observed trends, offering a unified and chemically intuitive framework to interpret the results.

### High-throughput Screening of Tunable Optoelectronic Materials

A high-throughput screening of crystalline materials was performed using the computational phonon database PhononDB.^20^ This database contains phonon calculations based on finite-displacement methods carried out with the PhonoPy software package.^21,22^ The database includes 10,034 distinct crystal structures, all of which originate from the materials project (MP),^23^ thereby enabling straightforward access to complementary material properties such as electronic band gaps.

Figure 1 outlines the screening workflow and materials selection criteria adopted in this study. The first step involved identifying compounds with centrosymmetric crystal structures, that is, possessing inversion symmetry. The rationale for focusing on centrosymmetric materials was two-fold. First, introducing phonon-like distortions in these highly symmetric structures can lift electronic band degeneracies, a well-known mechanism for modifying band gaps.^24^ Second, centrosymmetric systems can host polar phonon distortions that break inversion symmetry and may be externally activated by electric fields. This may allow for controlled and efficient tuning of optoelectronic properties via field-induced lattice distortions. A total of 7449 materials met this symmetry criterion and advanced to the next stage of the screening process.

**Figure 1:**
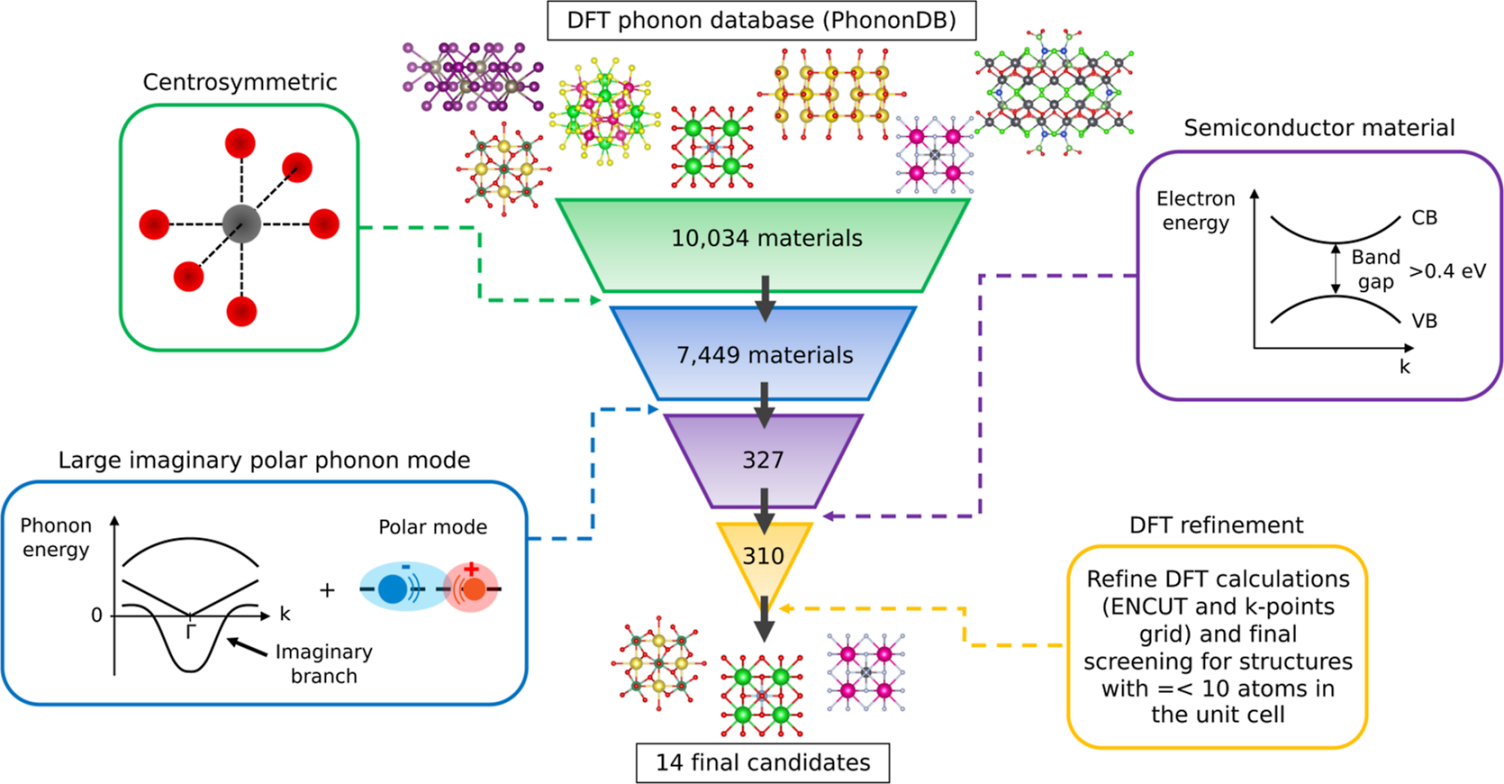
Representation of the adopted materials screening strategy. The screening process started with 10,034 materials and ended up with 14 final candidates. Structures were filtered by their crystal symmetry, Γ phonon modes, and band gap. Refined DFT calculations were conducted for 310 candidates with 10 atoms or less per unit cell. The atomic structure of some illustrative materials are represented.

As stated in the Introduction, our focus is on anharmonic materials due to their tendency to host low-energy optical phonon modes. These modes typically involve large atomic displacements and are likely to induce strong electron–phonon coupling.^15–17^ Although the PhononDB database contains only harmonic phonon calculations, it is still possible to infer signatures of anharmonicity from them. One such indicator is the presence of imaginary phonon modes.

Strongly anharmonic materials, while dynamically stable at finite temperatures, may exhibit imaginary frequencies in phonon dispersion relations calculated at zero temperature.^25^ Based on this rationale, our next screening criterion selected materials with at least one imaginary phonon frequency below 1.5i THz at the Γ-point. This frequency threshold was chosen to avoid the inclusion of spurious imaginary modes arising from the neglect of long-range multipolar interactions.^26^ For simplicity, we restricted our analysis to Γ-point phonons, although materials exhibiting imaginary modes elsewhere in the Brillouin zone may be also promising. Anharmonicity descriptors, like phonon line widths, could provide further insight; however, direct anharmonic calculations are computationally prohibitive for the present high-throughput screening study.

Furthermore, we required the aforementioned imaginary Γ-point phonon modes to be polar, that is, it should involve a net displacement between positively and negatively charged ions, thereby generating an electric dipole.^27^ Such polar modes are capable of coupling to external electric fields, hence are essential for applications.^28^ Applying this additional criterion narrowed the list to 327 candidate materials. We then further reduced the data set by retaining only semiconductor materials with a nonzero band gap. Band gap values were obtained from the MP,^23^ and materials with band gaps smaller than 0.4 eV were excluded to ensure well-defined semiconductor behavior. Of the initial 327 candidates, 310 compounds met this criterion. These materials, along with their MP identity number, chemical formula, space group, number of atoms in the unit cell, and band gap, are listed in the Supplementary files.

To refine those theoretical first-principles results, we recomputed selected properties using higher–precision parameters than employed in the PhononDB^20^ and MP^23^ databases (Methods). However, many of the shortlisted structures contained a large number of atoms per unit cell, ranging from dozens to hundreds, making such recalculations computationally very demanding. To ensure feasibility, we restricted this final refinement step to structures with ten or fewer atoms per unit cell, resulting in a subset of 24 candidates. For these compounds, we performed higher-accuracy geometry relaxations and Γ-point phonon calculations using DFT. The updated values are provided in Supplementary Table S1. Of the 24 materials, 14 continued to meet all the previous screening criteria following this refinement.

Among the 14 shortlisted candidates, 10 exhibit perovskite-like structures with the general formula ABX_3_ and adopt the typical paraelectric high-temperature cubic phase with space group *Pm*3̅*m*,^29^ illustrated in Figure 2a. The remaining four materials include one compound with a perovskite-related structure in the *P*4/*mbm* space group and three ternary compounds with *P*2_1_/*m* and *Pnnm* symmetries. To this list, we added four chalcohalide antiperovskite compounds, namely, Ag_3_SBr, Ag_3_SI, Ag_3_SeBr, and Ag_3_SeI, previously investigated in works^9,30,31^ although not present in the PhononDB database. These compounds were considered *a* posteriori due to their marked anharmonic behavior and strong electron–phonon coupling.^9^ Like the ABX_3_ perovskites, they adopt a cubic structure with *Pm*3̅*m* symmetry at finite temperatures.^30,31^ This addition brings the total number of analyzed materials to 18. Table 1 summarizes these candidates, reporting their chemical composition, space group, energy of the most unstable (imaginary) phonon mode, and band gap calculated at zero temperature.

**Figure 2:**
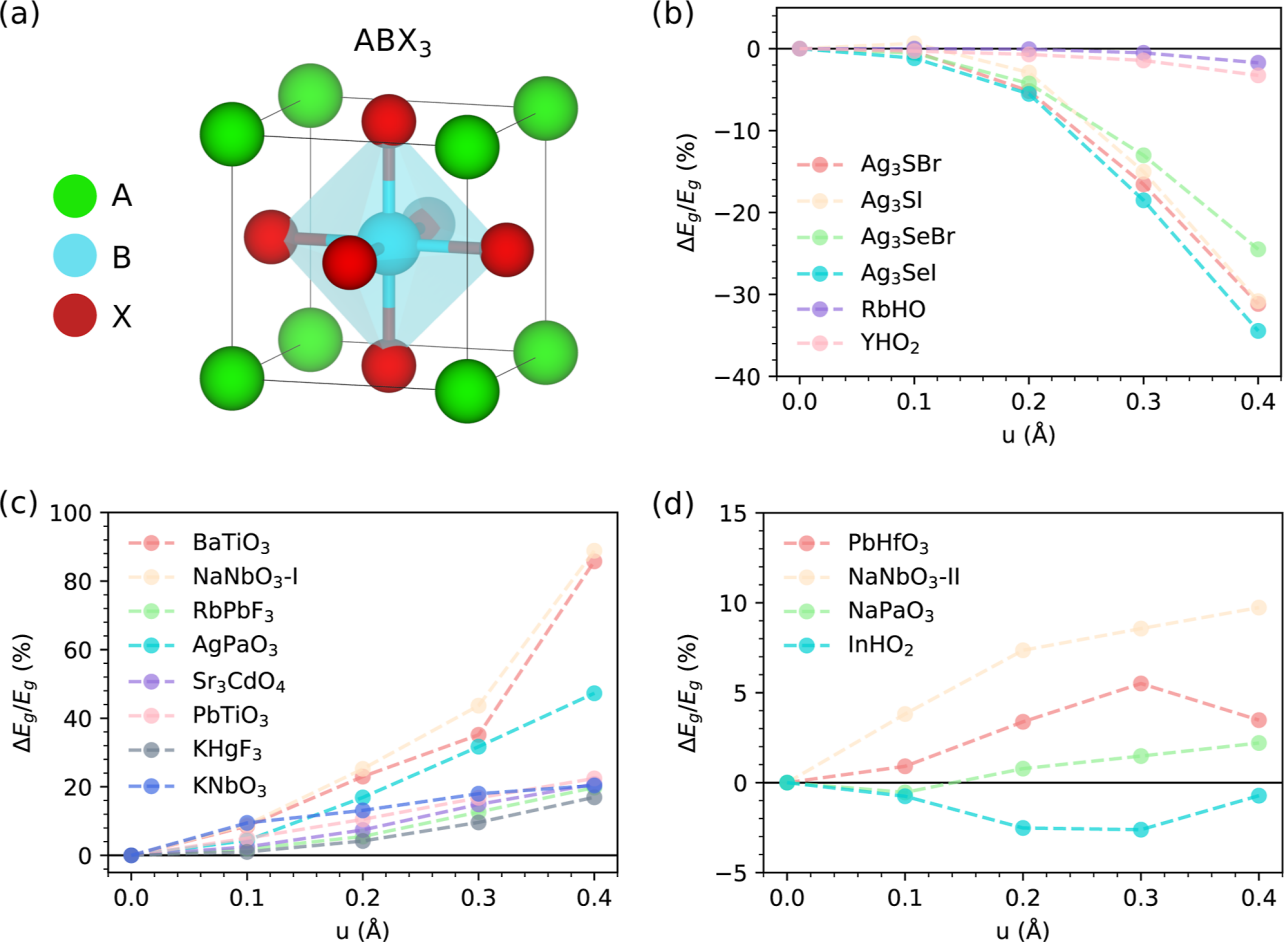
Perovskite-like structure and relative variation of the band gap under frozen-phonon distortions. (a) Perovskite and antiperovskite structures: B atoms are cations in perovskites and anions in antiperovskites; X atoms are anions in perovskites and cations in antiperovskites. Candidate materials grouped by phonon-driven band gap variation: (b) reduction, (c) increase, and (d) minimal change. NaNbO_3_–I and II correspond to two different polymorphs of the same compound with *Pm*3̅*m* and *P*4/*mbm* symmetries, respectively.

**Table 1:** Optoelectronic Tunable Materials Resulting from Our High-throughput Screening^a^

material	space group	*E*_Γ_	*E*_g_^0K^
		(meV)	(eV)
Ag_3_SBr	*Pm*3̅*m*	9.00i	1.8
Ag_3_SI	*Pm*3̅*m*	9.12i	1.4
Ag_3_SeBr	*Pm*3̅*m*	9.57i	1.6
Ag_3_SeI	*Pm*3̅*m*	9.16i	1.3
PbHfO_3_	*Pm*3̅*m*	15.09i	3.2
PbTiO_3_	*Pm*3̅*m*	14.66i	2.3
BaTiO_3_	*Pm*3̅*m*	16.75i	2.5
KNbO_3_	*Pm*3̅*m*	24.43i	2.4
NaNbO_3_–I	*Pm*3̅*m*	21.36i	2.5
NaNbO_3_–II	*P*4/*mbm*	18.88i	2.5
KHgF_3_	*Pm*3̅*m*	8.39i	1.8
RbPbF_3_	*Pm*3̅*m*	10.41i	3.6
NaPaO_3_	*Pm*3̅*m*	11.24i	4.5
AgPaO_3_	*Pm*3̅*m*	8.85i	1.5
Sr_3_CdO_4_	*Pm*3̅*m*	6.05i	1.6
YHO_2_	*P*2_1_/*m*	23.17i	5.5
InHO_2_	*Pnnm*	20.36i	3.2
RbHO	*P*2_1_/*m*	41.95i	4.7

a Summary of the structural, vibrational and band-gap properties of the 18 candidate compounds. *E*_Γ_ represents the (imaginary) energy of the largest Γ–point polar phonon instability, and *E*_g_^0K^ the band gap calculated at zero temperature.

### Band Gap Change Induced by Low-Energy Optical Polar Phonon Displacements

For each of the 18 sieved materials, we applied unit-cell structural distortions along the eigenvectors of their most unstable polar Γ phonon modes (i.e., those with the largest imaginary frequencies). The total displacement amplitude ranged from zero to 0.4 Å, and for each distorted configuration we computed the band gap, *E*_g_ (Methods). The relative change in *E*_g_ as a function of the distortion amplitude is presented in Figure 2b–d, where materials are classified into three different categories: monotonic decrease (Figure 2b), monotonic increase (Figure 2c), and other trends such as nonmonotonic or modest band gap variation (Figure 2d). It is important to emphasize that the maximum distortion amplitude of 0.4 Å has been arbitrarily chosen and that, while physically plausible, may not correspond to actual thermal displacements. We will revisit and discuss this important caveat in the next section.

In Figure 2b, the chalcohalide antiperovskites show a pronounced reduction in band gap under polar phonon distortion, in agreement with previous results.^9^ Notably, Ag_3_SBr and Ag_3_SI exhibit band gap decreases of approximately 30% at a distortion amplitude of 0.4 Å. In contrast, compounds such as RbHO and YHO_2_ display much weaker responses, with band gap reductions of less than 4% at the same displacement amplitude. Although these latter materials were initially identified as promising candidates for strong electron–phonon coupling, our specific analysis indicates that their actual tunability may be limited.

Figure 2c displays materials that exhibit a pronounced and steady increase in *E*_g_ as the phonon distortion amplitude grows. All these materials adopt perovskite-like structures with *Pm*3̅*m* symmetry. Compounds such as KHgF_3_ and PbTiO_3_ show significant band gap increases of approximately 20% for a maximum distortion amplitude of 0.4 Å. Remarkably, BaTiO_3_ and NaNbO_3_–I exhibit exceptional enhancements of nearly 100% under the same conditions.

Figure 2d presents the remaining materials, which do not exhibit monotonic or significant phonon-induced *E*_g_ variations. InHO_2_ displays a mild band gap reduction, whereas the other compounds, also perovskite-like, show only modest band gap increases (less than 10%) under the maximum considered phonon distortion. A noteworthy case is NaNbO_3_, which demonstrates that phonon-induced band gap variations can strongly depend on the specific polymorph. In particular, the *Pm*3̅*m* phase (referred to as polymorph I) exhibits a pronounced phonon-mediated band gap modulation approximately nine times greater than that of the *P*4/*mbm* phase (polymorph II) at the highest amplitude considered (Figure 2c,d).

It is worth noting that we do not find a correlation between the (imaginary) energy of the largest polar Γ-point phonon instability (*E*_Γ_ in Table 1) and the magnitude of the associated phonon-induced band gap variation. For example, RbHO exhibits the largest *E*_Γ_ among all candidates, yet shows only minimal *E*_g_ modulation (Figure 2b). In contrast, Sr_3_CdO_4_ has the smallest *E*_Γ_, yet displays an appreciable change in *E*_g_ (Figure 2c). These observations show that *E*_Γ_ alone is not a reliable descriptor for identifying materials with strong band gap tunability under polar phonon distortions.

### Representative Materials: Ag_3_SBr, BaTiO_3_ and PbHfO_3_

We selected three representative materials for in-depth analysis, each exemplifying one of the distinct trends in band gap behavior: Ag_3_SBr, which exhibits a strong band gap reduction; BaTiO_3_, which shows a pronounced band gap increase; and PbHfO_3_, which displays minimal variation. The corresponding crystal structures are depicted in Figure 3a–c. In conventional perovskites such as BaTiO_3_ and PbHfO_3_, anions (i.e., oxygen atoms) occupy the corners of the octahedra, with one type of cation residing at the octahedral center and another at the cube corners. In contrast, in the antiperovskite Ag_3_SBr, these roles are inverted: the octahedral corners are occupied by cations (i.e., silver atoms), while anions reside at the center and corner positions.

**Figure 3:**
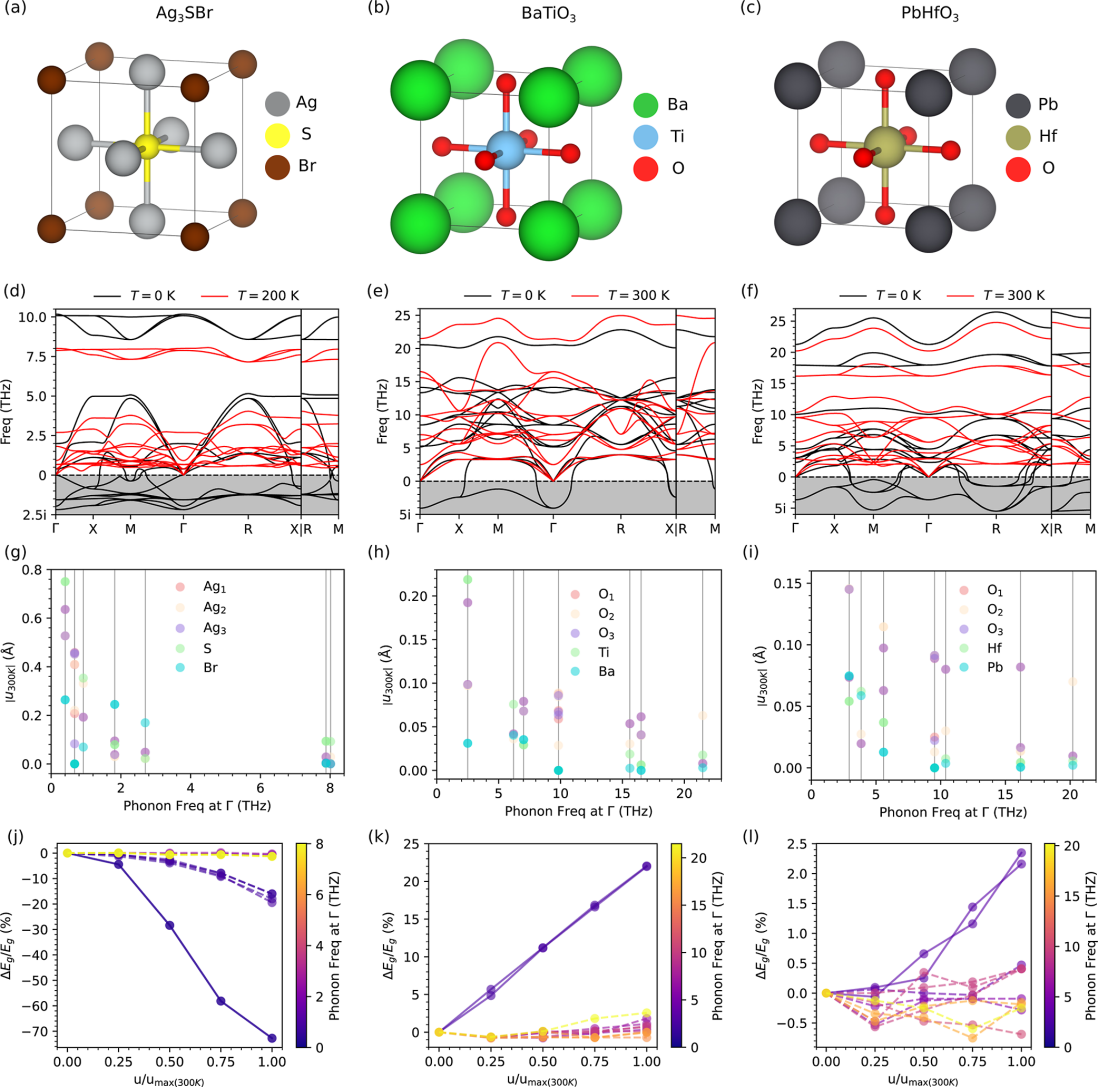
Phonon dispersions and band gap relative variation for representative materials. (a–c) Unit cell structure of Ag_3_SBr (band gap decreases), BaTiO_3_ (increases), and PbHfO_3_ (minimal change). (d–f) Harmonic (black) and *T*-renormalized (red) phonon dispersions. (g–i) Atomic displacements corresponding to phonon distortions calculated at finite temperature. Vertical lines indicate phonon energies at the Γ-point. (j–l) Band gap changes induced by Γ-point phonon distortions, scaled by the maximum amplitude obtained at finite temperature. Lines are guides to the eye; two key low-energy optical modes are represented with solid lines.

The high-symmetry cubic *Pm*3̅*m* phase in perovskite-like structures is known to be vibrationally unstable at *T* = 0 K. However, it can be dynamically stabilized at finite temperatures due to thermal lattice effects.^32–34^ This cubic polymorph is often observed at moderate and high temperatures as a result of phase transitions from lower-symmetry structures, typically involving distortions or tilting of the anion octahedra.^35,36^ To characterize and analyze the vibrational behavior of the *Pm*3̅*m* phase, we computed the phonon spectra of Ag_3_SBr, BaTiO_3_, and PbHfO_3_ at both *T* = 0 and 300 K (Figure 3d–f, Methods). For Ag_3_SBr, finite-temperature calculations were conducted at 200 K to avoid computational bias arising from superionicity.^31^ At zero temperature, all three compounds exhibit several optical phonon branches with imaginary frequencies, confirming their dynamic instability in this limit. However, upon incorporating thermal effects (Methods), these instabilities are removed, in agreement with experimental observations at ambient and high-*T* conditions.

For a Γ-point phonon mode indexed by ν, the maximum amplitude of the vibrational displacement for atom *j* along the α Cartesian direction is given by^22^

1 $$u_{j,\nu }^{\alpha }=\sqrt{\frac{\hbar }{2m_{j}\omega_{\nu }}}\sqrt{1+2n_{\nu }\left(T\right)}\left|e_{j,\nu }^{\alpha }\right|$$

where ω_ν_ is the mode frequency, *m*_*j*_ is the mass of atom *j*, *n*_ν_(*T*) is the Bose–Einstein occupation factor at temperature *T*, and **e**_*j*,*ν*_^α^ is the normalized eigenvector component of the phonon mode. From this expression, it follows that atomic displacements become larger for lighter atoms, low-frequency modes, and elevated temperatures. This behavior is clearly shown in Figure 3g–i, where we depict the magnitude of the atomic displacements associated with Γ-point phonons at *T* = 300 K (made the exception of Ag_3_SBr, for which finite-temperature calculations were conducted at 200 K^31^).

Ag_3_SBr, which features ultra low-energy optical phonon modes around 0.4 THz, exhibits exceptionally large atomic displacements: up to 0.8 Å for S atoms and 0.6 Å for Ag atoms (Figure 3g). In contrast, BaTiO_3_ and PbHfO_3_, whose lowest-energy phonon modes lie at approximately 2.5 and 3.0 THz respectively, display significantly smaller, though still considerable, displacements: 0.1–0.2 Å for BaTiO_3_ (Figure 3h) and 0.05–0.15 Å for PbHfO_3_ (Figure 3i). For higher-energy phonons, the atomic displacements are further suppressed, remaining below 0.2 Å in Ag_3_SBr and below 0.1 Å and 0.05 Å in BaTiO_3_ and PbHfO_3_, respectively. These findings indicate that Ag_3_SBr is likely more anharmonic than the two perovskites, owing to its larger vibrational amplitudes and, consequently, offers greater potential for lattice-mediated electronic effects.

Figure 3j–l present the relative band gap variation with respect to the equilibrium (undistorted) structure, using phonon-mode displacements with maximum amplitudes computed at *T* = 300 K via eq 1. In Ag_3_SBr (Figure 3j) and BaTiO_3_ (Figure 3k), we observe that low-energy phonon modes are primarily responsible for substantial band gap changes: up to a 70% reduction in Ag_3_SBr and more than a 20% increase in BaTiO_3_ at the corresponding maximum displacements. In Ag_3_SBr, low-frequency lattice vibrations strongly reduce the band gap, whereas higher-frequency modes have a negligible effect. In BaTiO_3_, low-energy modes markedly increase the band gap, while higher-energy modes produce minor changes, with some even slightly decreasing the band gap. The smaller size of the band gap enhancement in BaTiO_3_ compared to that shown in Figure 2c arises from the reduced phonon displacements calculated at *T* = 300 K, which are all smaller than 0.4 Å.

It is important to note that as temperature increases, the relative influence of phonon modes changes due to the Bose–Einstein occupancy distribution: low-energy phonons become less dominant, while higher-energy modes gain increasing weight (Supplementary Figure S1 and Supplementary Discussion). Consequently, even if low-energy phonons initially drive a band gap increase or decrease at low temperatures, a compensating, or even opposite, effect may emerge at higher temperatures, leading to *T*-induced nonmonotonic band gap behavior. Examples of semiconductors, not considered in this study, where the band gap first increases with temperature before decreasing include chalcopyrites^12^ and single-walled carbon nanotubes.^39^

In the case of PbHfO_3_ (Figure 3l), phonon-mode distortions generally lead to minimal changes in the band gap, many of which fall within the typical accuracy limit of our DFT calculations (∼0.1 eV). Nevertheless, the low-energy modes exhibit a consistent, albeit modest, tendency to increase the band gap by approximately 2.5% at 300 K. In contrast, specific higher-energy modes exert a weak decreasing effect. This indicates a temperature-driven competition between phonon modes that slightly increase and others that slightly decrease the band gap. As a result, even though PbHfO_3_ may undergo sizable atomic displacements due to anharmonicity and thermal effects, its net band gap variation remains small. As we will discuss later, this limited tunability is closely linked to the specific nature of electronic orbital hybridizations in this compound.

### Band-Gap Dependence on Temperature

We computed the thermally renormalized band gaps of the three representative materials, Ag_3_SBr, BaTiO_3_ and PbHfO_3_, using the methodology described in the Methods section and in work.^9^ Calculations were performed at *T* = 300 and 600 K, accounting for both short-range and long-range electron–phonon contributions. Long-range contributions, which stem from limitations associated with the use of finite supercell sizes in simulations of polar materials, were corrected using the Fröhlich polaron approach^40,41^ (Methods). Short-range contributions, were accounted for using finite-difference methods^42^ (Methods). Quantum nuclear effects were systematically neglected since are expected to be small in materials composed of heavy atoms,^43^ thus significantly improving computational efficiency. Our results are summarized in Table 2 and compared with available experimental room-temperature data from the literature.

**Table 2:** Temperature-Dependence of the Calculated Band Gaps^a^

material	*E*_g_^0K^(eV)	*E*_g_^300K^(eV)	Δ*E*_g_^S^(meV)	Δ*E*_g_^L^(meV)	*E*_g_^600K^(eV)	Δ*E*_g_^S^(meV)	Δ*E*_g_^L^(meV)	*E*_*g*_^*exp*^(eV)
Ag_3_SBr	1.8 ± 0.1	1.2 ± 0.1	–480	–74	0.9 ± 0.2	–680	–175	1.0 ^30^
BaTiO_3_	2.5 ± 0.1	3.5 ± 0.1	+1060	–38	3.1 ± 0.1	+770	–160	3.2 ^37^
PbHfO_3_	3.2 ± 0.1	3.4 ± 0.1	+220	–65	2.9 ± 0.1	–50	–230	3.4 ^38^

a *E*_g_ values were obtained at zero temperature (excluding quantum nuclear effects), 300 K, and 600 K. Short- and long-wavelength phonon band-gap corrections, Δ*E*_g_^S^ and Δ*E*_g_^L^ (Methods), are provided at each temperature. Numerical uncertainties are provided, mainly resulting from the Δ*E*_g_^S^ correction term. Experimental room-temperature band gaps for Ag_3_SBr,^30^ BaTiO_3_ ^37^ and PbHfO_3_,^38^ are shown for comparison.

We found excellent agreement between our calculated finite-temperature band gaps and experimental room-temperature data for the three considered materials (Table 2). For Ag_3_SBr and BaTiO_3_, there exists a substantial discrepancy of approximately 0.8 eV between the computed zero-temperature band gaps and the corresponding experimental room-temperature values: the theoretical *E*_g_ of Ag_3_SBr is considerably larger than the experimental one, whereas that of BaTiO_3_ is significantly smaller. However, when the band gaps are computed at *T* = 300 and 600 K, we observe a pronounced reduction in *E*_g_ for Ag_3_SBr and an increase for BaTiO_3_, leading to excellent agreement with the experimental data within the numerical uncertainties. In the case of PbHfO_3_, the zero-temperature band gap is already in good agreement with the experiments, with a deviation of only ∼0.1 eV.

For Ag_3_SBr, both short- and long-range electron–phonon interactions contribute to the reduction of the band gap. While short-range effects dominate, being approximately 4–6 times larger than the long-range contributions, the latter become increasingly significant as temperature rises. At *T* = 300 K, the band gap is reduced by approximately 33% relative to its zero-temperature value, indicating a giant thermal renormalization effect, consistent with the findings reported in work.^9^ At *T* = 600 K, the band gap is further reduced to nearly half of its static value, underscoring the pronounced role of electron–phonon coupling in determining the finite-temperature electronic properties of this material.

For BaTiO_3_, we observe a pronounced band gap increase primarily driven by short-range phonon contributions. At *T* = 300 K, the short-range correction enhances the band gap by nearly 1.0 eV. At *T* = 600 K, this positive contribution slightly diminishes, yielding an increase of approximately 0.8 eV relative to the zero-temperature value. As mentioned earlier, as temperature rises higher-energy phonon modes gain importance and, since these modes tend to reduce the band gap (Figure 3k), the overall temperature dependence is nonmonotonic. The long-range Fröhlich contribution is comparatively minor, resulting in a consistent band gap reduction of 0.04 eV at *T* = 300 K and 0.2 eV at 600 K.

For PbHfO_3_, the long-range phonon contribution surpasses the short-range component at elevated temperatures, resulting in a modest overall reduction of the band gap (Figure 3l). At *T* = 300 K, short-range interactions increase the band gap by approximately 0.2 eV, whereas long-range effects reduce it by 0.07 eV. At *T* = 600 K, the total variation in the band gap remains below 10%, driven by a more pronounced long-range reduction of 0.2 eV and a concurrent, though smaller, short-range decrease of 0.05 eV.

From this analysis, we conclude that Ag_3_SBr and BaTiO_3_ exhibit pronounced electron–phonon interactions that significantly influence their optoelectronic properties. As such, incorporating these effects is crucial for achieving accurate agreement with experimental measurements. In contrast, despite the strong anharmonicity of PbHfO_3_, electron–phonon interactions exert only a limited effect on its band gap. It is important to underscore that most band gap calculations reported in the literature are performed at *T* = 0 K, often neglecting thermal effects under the assumption that they are negligible. However, our results demonstrate that this assumption does not hold universally. Accurately capturing the temperature dependence of optoelectronic properties in semiconductors, especially in materials with soft phonon modes or strong lattice anharmonicity, requires explicit consideration of electron–phonon interactions.

It is worth noting that band gap renormalization effects have been analyzed in various semiconductors not considered in this study. For instance, CuCl exhibits a band gap increase of approximately 60 meV at room temperature^13^ while in the chalcopyrites ZnSnSb_2_ and CdGeAs_2_ the band gap decreases by about 150 and 40 meV, respectively, under the same conditions.^12^ Antimony sulfide (Sb_2_S_3_), a promising material for photovoltaic applications, also shows a substantial band gap reduction of nearly 200 meV at room temperature.^44^ Notably, our DFT results for Ag_3_SBr and BaTiO_3_ indicate significantly larger band gap renormalization effects at room temperature.

### Electron–Phonon Coupling Mechanisms

To elucidate why Ag_3_SBr and BaTiO_3_ exhibit strong but opposite band gap trends with increasing temperature, while PbHfO_3_ does not, we further analyzed their underlying electronic mechanisms using complementary tight-binding (TB) models. Our analysis focuses on the low-energy polar optical phonon modes, which dominate electron–phonon interactions at low temperatures.

Figure 4a–c display the electronic density of states (eDOS) near the valence band maximum (VBM) and conduction band minimum (CBM) for the three representative materials, as computed with DFT. In Ag_3_SBr, the valence band comprises a mixture of Ag-d, Br-p, S-p, and Ag-s orbitals. In contrast, the valence bands of the perovskites BaTiO_3_ and PbHfO_3_ are primarily dominated by O-p states. Regarding the conduction band, Ag_3_SBr features contributions from Ag-s, S-s, and Ag-d orbitals; BaTiO_3_ mainly from Ti-d and O-p; and PbHfO_3_ from Pb-p and O-p. These band-edge orbitals are crucial for understanding the distinct thermal band gap behaviors observed in these materials, as we elaborate next.

**Figure 4:**
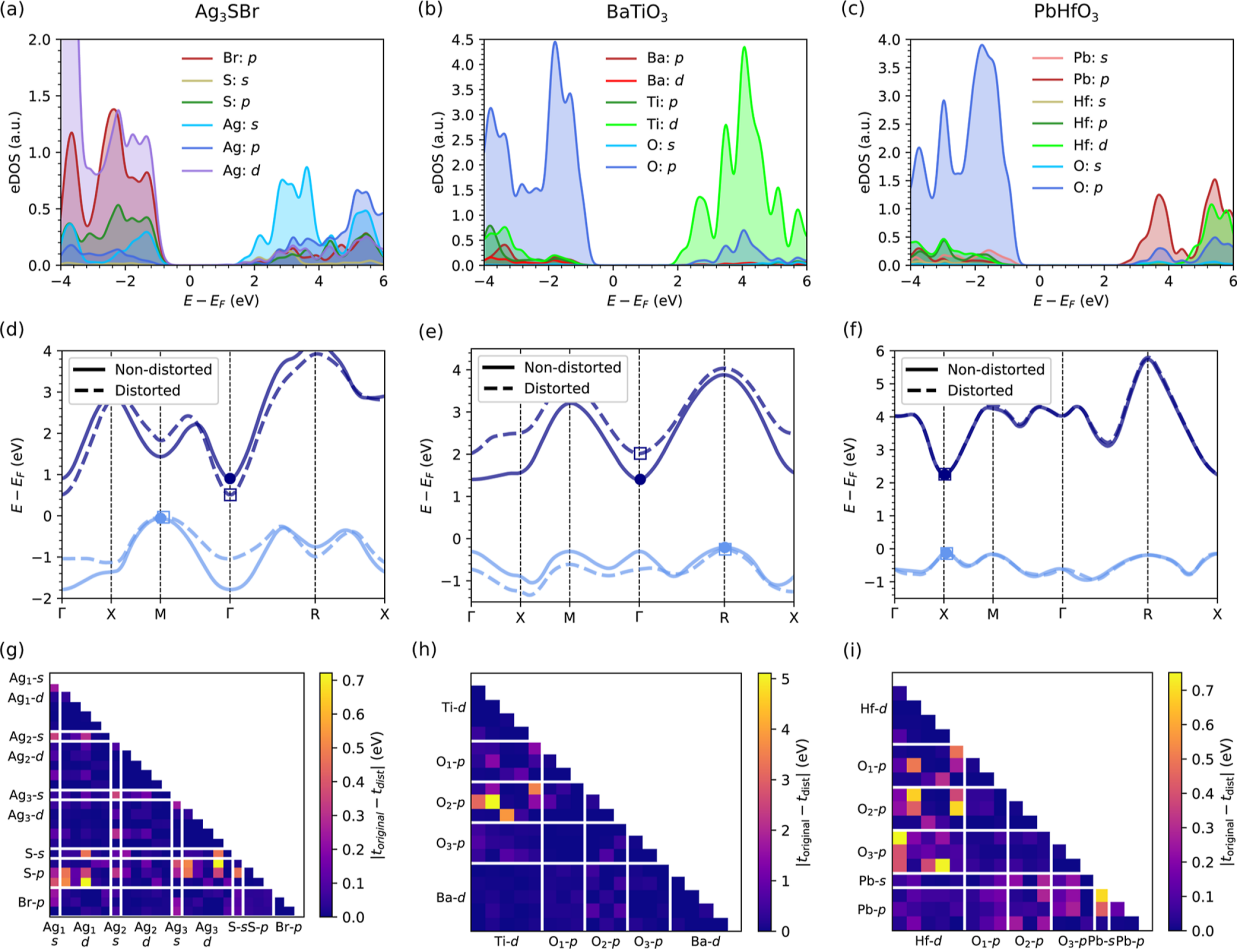
Electronic properties and band gap variation mechanisms for representative materials. (a–c) Density of states near the band gap for Ag_3_SBr, BaTiO_3_, and PbHfO_3_, showing contributions from the most relevant orbitals. (d–f) Valence band (light blue) and conduction band (dark blue) for the equilibrium (solid) and phonon distorted (dashed) structures. (g–i) Changes in tight-binding hopping terms for each compound under phonon distortion. Warmer colors indicate greater changes in orbital overlap; white lines separate orbital types. The Hamiltonian matrix is symmetric.

Figure 4d–f display the band structures calculated near the VBM and CBM for the three materials in both their equilibrium (undistorted, solid lines) and phonon-distorted (dashed lines) configurations. The distorted structures correspond to atomic displacements of 0.2 Å along the eigenvector of the lowest-energy optical phonon mode at the Γ-point. To reduce computational cost, these band structures were computed using a standard semilocal exchange–correlation functional. This approximation is justified because, while the absolute band gap values may be underestimated, the fundamental band-dispersion trends are well reproduced (Supplementary Figure S2).

In Ag_3_SBr, we observe that the CBM shifts to lower energies under the phonon distortion, while the VBM remains essentially unchanged and becomes slightly flatter, in agreement with prior work.^9^ The observed band gap reduction in Ag_3_SBr is therefore primarily driven by the downward shift of the CBM. In BaTiO_3_, the CBM shifts upward noticeably, while the VBM slightly moves downward. Consequently, the band gap increase in BaTiO_3_ results from both an upward shift of the CBM and a downward shift of the VBM. In contrast, PbHfO_3_ exhibits minimal changes, with only a barely detectable downward shift of the VBM, consistent with the small band gap increase reported in Figure 3l.

To gain deeper insight into the electronic mechanisms driving these trends, we constructed a TB model (Methods) to analyze how orbital hybridizations evolve under phonon distortion. Figure 4g–i show the changes in TB hopping parameters between different atomic orbitals before and after introducing the phonon distortion. In the TB formalism, the hopping parameters, which quantify the probability amplitude for an electron to hop from one atomic orbital to another, are given by the off-diagonal elements of the TB Hamiltonian, ⟨*n*|*H*|*m*⟩, where *n* and *m* denote electronic orbitals and *H* the TB Hamiltonian. These off-diagonal TB Hamiltonian elements are identified as kinetic energies. On the other hand, the diagonal terms of the Hamiltonian, ⟨*n*|*H*|*n*⟩, provide the on-site orbital energies. These diagonal TB Hamiltonian elements are identified as potential energies. When two orbitals hybridize, both a larger hopping term and a smaller on-site energy difference between them are associated with an increased energy splitting for the resulting bonding and antibonding states.

In Ag_3_SBr, the most notable, albeit still modest, changes in electronic hybridization occur between the S-p and Ag-d orbitals (Figure 4g). However, this orbitals hybridization does not contribute significantly to the CBM and therefore cannot account for the observed *E*_g_ trend. In contrast, we observe substantial changes in the difference between the on-site energies of the Ag-s and S-s orbitals before and after introducing the distortion. Supplementary Table S2 presents the numerical values for the relevant orbitals, showing that the hopping term increases slightly by 0.13 eV with the distortion, while the difference in potential energy between the Ag-*s* and S-s orbitals decreases by a total of 1.63 eV. This notable energy difference decrease leads to a pronounced energy lowering of the bonding state formed by the Ag-*s* and S-s orbitals. Since these bonding states contribute significantly to the CBM, their energy reduction results in a substantial narrowing of the band gap.^9^

In BaTiO_3_, the most significant changes in hybridization are observed between the O-p and Ti-d orbitals (Figure 4h). Phonon distortions notably increase the energy splitting between the bonding and antibonding states arising from this orbital hybridization. From Supplementary Table S2, we observe a substantial increase of 5.11 eV in the hopping term between the O-p and Ti-d orbitals. There is also a decrease in the difference between their on-site energies, although this is modest as it amounts to 0.52 eV. Since the bonding states lie at the VBM and the antibonding states at the CBM, this enhanced splitting leads to an opening of the band gap.

In PbHfO_3_, the primary hybridization change involves the O-p and Hf-d orbitals (Figure 4i). Although a bonding–antibonding splitting occurs, similarly to BaTiO_3_, the antibonding states lie at energies well above the CBM, and therefore do not significantly contribute to band gap variation. The slight upward shift in the VBM, driven by the O-p and Hf-d orbital hybridization, accounts for the modest band gap increase. Furthermore, the changes in hopping parameters are smaller than in BaTiO_3_, resulting in weaker orbital splitting and consequently a smaller overall effect on the band gap.

The concept that band gap variations can arise from the alignment of the VBM and CBM with bonding and antibonding states and from changes in their energy splitting, was previously investigated in work.^45^ However, while that study focused on how chemical doping in perovskite-like systems modifies orbital hybridization, and consequently *E*_g_, our work demonstrates that similar hybridization changes can also be induced by phonon distortions, specifically those associated with low-energy polar modes.

By examining the phonon dispersions (Figure 3d–f) in conjunction with the electronic band structures (Figure 4d–f), we can qualitatively assess the impact of electron–phonon coupling on the band gap. Taking BaTiO_3_ as a representative example, the Γ, *M*, and *R* points in the Brillouin zone are expected to contribute most significantly to the band gap modulation, given their proximity to the VBM and CBM. From the phonon dispersion in Figure 3e, we observe pronounced thermal renormalization at Γ and *M*, whereas the changes at *R* are comparatively modest. Accordingly, more substantial modifications to the electronic bands are anticipated at Γ and *M* than at *R*. This expectation is indeed corroborated by the valence and conduction band distortions observed in Figure 4e. A similar analysis applied to Ag_3_SBr (Figures 3d and 4d) leads to an analogous conclusion: the Γ and *M* points dominate the electron–phonon interactions responsible for thermal band gap renormalization.

### General Electron–Phonon Coupling Framework

To further rationalize and generalize the band gap variation under low-energy phonon distortions observed in Ag_3_SBr, BaTiO_3_, and PbHfO_3_, we focus on the archetypal nondistorted perovskite-like structure with symmetry group *Pm*3̅*m*, depicted in Figure 5a. In this structure, the atoms labeled B (center of the octahedron) and X (octahedral corners) are crucial in the orbital hybridizations that influence band gap variations. Figure 5b illustrates the general principle: hybridization between the B and X atom orbitals results in bonding and antibonding states; when the lattice is distorted by a polar phonon mode, the increased orbital overlap and the reduction between their difference in potential energies enhance the splitting between these states. Whether this splitting leads to an increase or decrease in the band gap depends on the positions of the bonding/antibonding states relative to the band edges, as well as the presence of other orbital contributions within the hybridization splitting.

**Figure 5:**
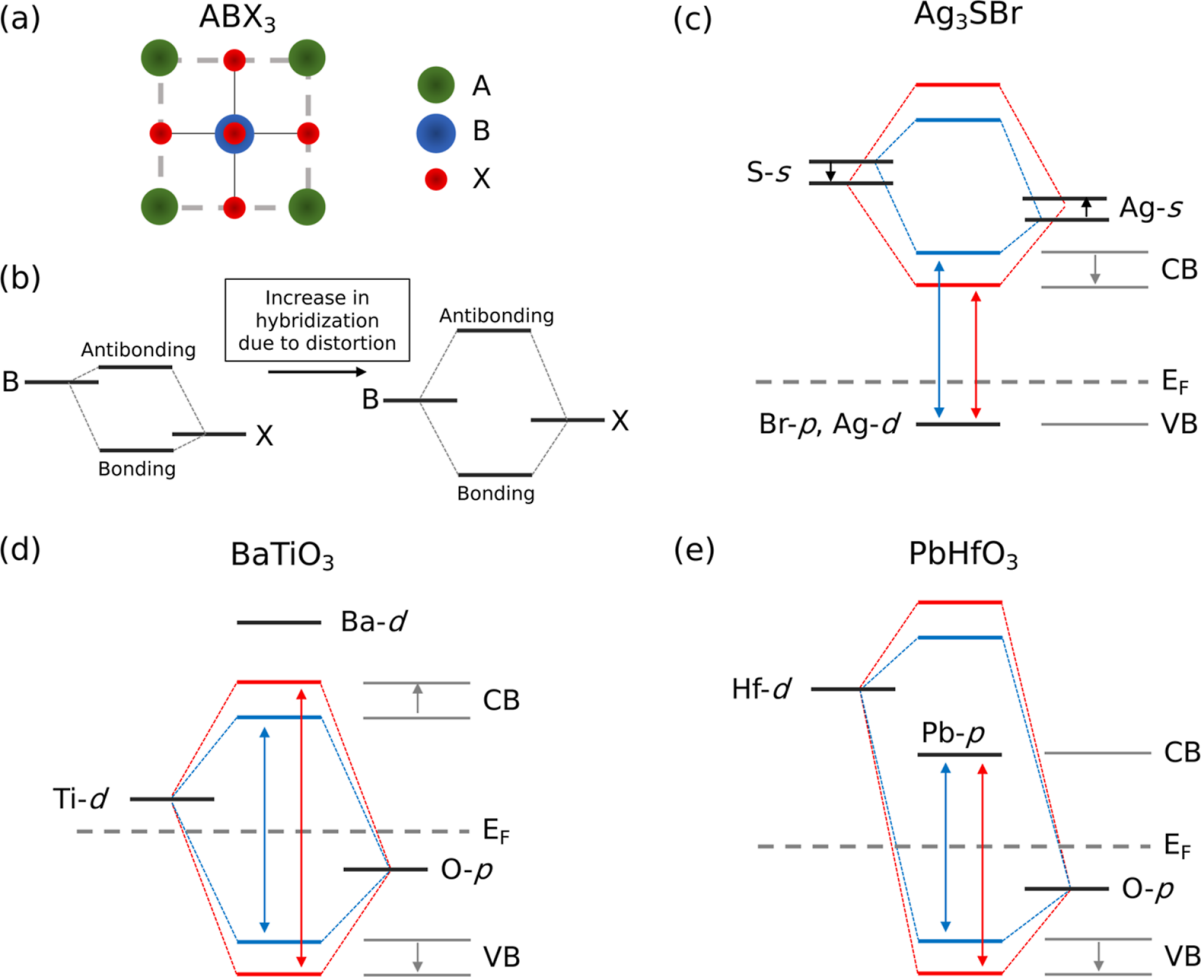
General electron–phonon coupling mechanisms driving temperature-induced band gap variation in perovskite-like semiconductors. (a) Equilibrium perovskite-like ABX_3_ structure. (b) Increased bonding–antibonding splitting from orbital hybridization due to a soft polar phonon mode distortion. (c) Band-gap reduction in Ag_3_SBr resulting from Ag–S s-orbitals hybridization and conduction band lowering. Band gap increase via hybridization in (d) BaTiO_3_ and (e) PbHfO_3_. In BaTiO_3_, bonding and antibonding states lie at the VBM and CBM, respectively. In PbHfO_3_, the antibonding state lies above the CBM hence phonon-induced splitting causes an almost negligible band gap change.

Figure 5c–e schematically illustrate the orbital hybridization scenarios for the three materials discussed in the previous sections. Changes in on-site energy are depicted only in Figure 5c, as they play a dominant role in lowering the bonding-state energy of this particular material. For the other two materials, such contributions are comparatively minor and therefore omitted. Numerical values of the relevant tight-binding (TB) matrix elements are reported in Supplementary Table S2.

Based solely on the eDOS of a given material, now we can explain why the different perovskite-like systems reported in Figure 2 exhibit distinct phonon-induced band gap behaviors. Supplementary Figure S3 presents the eDOS of the equilibrium structures for additional perovskite-like compounds identified in our screening, all with *Pm*3̅*m* symmetry. These include Ag_3_SeBr, Ag_3_SeI, Ag_3_SI, PbTiO_3_, NaNbO_3_, KNbO_3_, NaPaO_3_, AgPaO_3_, KHgF_3_, and RbPbF_3_. For the antiperovskite compounds reported in Figure 2b, we anticipate a similar band gap reduction mechanism as observed in Ag_3_SBr, given their eDOS similarity (Supplementary Figure S3a–c).

For PbTiO_3_, NaNbO_3_, and KNbO_3_ (Supplementary Figure S3d–f), the scenario closely resembles that of BaTiO_3_. Their VBM are dominated by O-p orbitals, while the CBM primarily consist of B-site d electrons. Consequently, phonon-induced distortions are expected to enhance the hybridization between the B-d and O-p orbitals, resulting in a widening of the band gap. This prediction aligns with the positive band gap shifts reported in Figure 2. Likewise, NaPaO_3_ and AgPaO_3_ exhibit an eDOS similar to that of BaTiO_3_, although in these systems the relevant orbital hybridizations occur between O-p and Pa-f states.

For KHgF_3_ and RbPbF_3_ (Supplementary Figure S3i,j), the band gap increase can also be interpreted using the hybridization mechanism discussed for BaTiO_3_. In both materials, the valence and conduction bands are primarily composed of X-site (F) and B-site (Hg or Pb) orbitals, likely forming bonding and antibonding states. Upon phonon distortion, the increased splitting of these states results in a larger band gap.

It is worth noting that the electronic behavior of materials not explicitly analyzed in this study but which adopt the perovskite structure and involve similar atomic species (e.g., ternary oxides) can likely be understood using arguments analogous to those presented here. A representative example is the well-known perovskite SrTiO_3_. The experimentally measured band gap of this compound at room temperature is approximately 3.3 eV,^46^ whereas standard DFT calculations typically predict significantly lower values. In our zero-temperature calculations (Supplementary Table S1), the band gap is found to be 2.7 eV, that is, 0.6 eV below the experimental result. Given the similarity in eDOS between SrTiO_3_ and BaTiO_3_, it is reasonable to expect that electron–phonon coupling also plays a significant role in SrTiO_3_, potentially accounting for the discrepancy between static DFT predictions and experimental observations.

## Discussion

We have demonstrated that electron–phonon corrections to the band gap in perovskite-like systems can be effectively explained through a combination of phononic and electronic coupled mechanisms. The band gap change is directly linked to distortions induced by polar lattice vibrations, which alter the hybridization between electronic orbitals. This hybridization reshapes the energy landscape of the electronic bands, and when these changes impact the extrema of the valence and conduction bands, the band gap is modified. A central factor in this optoelectronic modulation mechanism is anharmonicity, which is associated with low-energy optical modes capable of producing large atomic displacements. Nevertheless, as illustrated by PbHfO_3_, anharmonicity alone does not guarantee strong band gap renormalization; effective coupling between specific phonon modes and electronic states near the band edges is essential.

The polar character of the phonon modes under study is particularly significant, as it implies they can be externally excited using electric fields, rather than relying solely on thermal activation. This opens the door to potential applications in optoelectronic devices where the band gap, and more broadly the electronic and optical properties, can be dynamically tuned with electric fields.

To evaluate the practical feasibility of using electric fields to tune the optoelectronic properties of semiconductors, we estimated the order of magnitude of the electric fields required to excite relevant polar phonon modes to target amplitudes (Supplementary Table S3 and Supplementary Discussion).^47^ For Ag_3_SBr, the electric field needed to induce a soft polar mode distortion of 0.8 Å was found to be as low as 0.3 kV/cm, that is, well below the typical experimental range of 1–10 kV/cm. Similarly, for BaTiO_3_, an electric field of 0.8 kV/cm is sufficient to produce a distortion of 0.4 Å. In contrast, PbHfO_3_ requires higher, yet still experimentally accessible, fields, up to 1.5 kV/cm to induce a distortion of 0.2 Å. (However, as previously discussed, this material is considered less promising due to its weak electron–phonon coupling.) Overall, these results support the high practical feasibility of the proposed strategy for electric-field-driven optoelectronic tuning.

In this work, we have primarily focused on band gap changes, however electron–phonon interactions can influence the entire electronic structure and, consequently, the optical response of the material. For instance, previous research on chalcohalide antiperovskites has demonstrated a significant increase in the absorption coefficient with temperature, making these materials promising for photovoltaic applications.^9^ Low-energy optical nonpolar modes may similarly lead to significant electron–phonon effects, although since they cannot be excited via electric fields were neglected in this study. Nevertheless, with advances in laser technology and time-resolved spectroscopy, it is now feasible to excite nonpolar modes using light sources,^48–50^ broadening the range of tools available for efficient control or functionality.

Our screening identifies perovskite-like systems as the most promising candidates for exhibiting large optoelectronic tunability. Nonetheless, other material families not covered in our high-throughput study may also hold potential. Exploring these possibilities will require broader computational screenings that go beyond the Γ-point, incorporating phonon contributions across the entire Brillouin zone, and including materials beyond those currently available in the PhononDB database.^20^ Encouragingly, the rapid advancement of machine-learned interatomic potentials (MLIPs),^51^ such as M3GNet^52^ and MACE,^53^ offers a promising route toward scalable, high-throughput modeling of vibrational properties. These methods allow for accurate force predictions and molecular dynamics simulations at a fraction of the computational cost of DFT, with steadily improving accuracy.^54^ As a result, MLIPs can be effectively leveraged to expand the scope and scale of existing materials databases, enabling more comprehensive exploration of tunable optoelectronic materials.

Solid solutions are also expected to exhibit significant electron–phonon interactions, depending on the choice of parent compounds and their structural and optoelectronic similarities. However, additional effects not considered in this study, such as band gap bowing, can arise as the solid-solution composition varies.^30,55^ Based on the insights gained from our analysis, previously synthesized solid solutions such as Ag_3_SBr_*x*_I_1–*x*_ ^30^ and Na_*x*_K_1–*x*_NbO_3_ ^56^ are likely to exhibit strong electron–phonon coupling. Incorporating alloyed compounds considerably broadens the range of potentially promising materials for tunable optoelectronic applications. Addressing the inherent complexity of such systems will benefit significantly from advanced machine learning methods and tools.^57^ The authors are actively pursuing this line of computational materials research.

Finally, it is worth highlighting that some of the candidate materials identified in this study have already been synthesized and implemented in optoelectronic devices. For example, Ag_3_SBr and BaTiO_3_ have been incorporated into various solar cell components.^30,58–60^ Nevertheless, important challenges remain. In particular, current chemical synthesis methods for Ag_3_SBr are not yet scalable for industrial production, and ensuring thermal stability is essential to preserve the desired structural phase over the relevant operational temperature range. Further research is therefore required to overcome these limitations and to evaluate the practical feasibility of electric-field-induced band gap tuning in these promising materials.

## Conclusions

From our screening of approximately 10,000 materials, we identified several hundred candidates with significant band gap changes driven by low-energy polar phonon modes, enabled by strong electron–phonon coupling. We validated this behavior in a subset of perovskite-like systems, presenting and generalizing both phononic and electronic mechanisms to explain the diverse electron–phonon renormalization effects found on the materials optoelectronic properties. Additionally, we supported our theoretical findings for temperature-induced band gap variations with experimental evidence, confirming the accuracy of our approach and explaining possible discrepancies between ambient experimental results and zero-temperature computational predictions.

This work not only advances the fundamental understanding of electron–phonon interactions in perovskite-like materials, through clear and intuitive physical and chemical reasoning, it also lays the theoretical groundwork for leveraging these strong renormalization effects in future technological applications. These findings open the possibility of dynamically tuning the optoelectronic properties of semiconductors using electric fields, temperature, or light, offering exciting opportunities for next-generation optoelectronic devices.

## Methods

### Zero-Temperature First-Principles Calculations

DFT calculations^43,61^ were performed with the VASP software^62–64^ and semilocal PBEsol exchange–correlation functional.^65^ Wave functions were represented in a plane-wave bases set truncated at 700 eV. We selected a dense k-point grid, with 8 × 8 × 8 points for the reciprocal-space Brillouin zone (BZ) sampling, for the cubic perovskite systems. We obtained zero-temperature energies converged to within 0.5 meV per formula unit. For geometry relaxations, a force tolerance of 0.005 eV  Å^–1^ was imposed in all the atoms. The electronic bands and electronic density of states were estimated using the hybrid functional HSEsol and considering spin–orbit coupling (SOC) effects.^66,67^ Supplementary Table S4 reports the static (*T* = 0 K) band gaps of Ag_3_SBr calculated using various DFT exchange–correlation functionals: the semilocal PBEsol and PBE,^65,68^ the hybrid HSE06 and HSEsol,^66,67^ and the meta-GGA SCAN.^69^ For each case, calculations were performed both with and without including SOC effects. Although the inclusion of SOC results in only a modest change, it consistently reduces the band gap by approximately 0.1 eV across all functionals. The use of HSEsol with SOC is consistent with established practices in the computational study of optoelectronic materials, as this combination is widely employed for accurate band gap predictions.^70,71^ Quantum nuclear effects^43^ were disregarded throughout this work.

### Finite-Temperature First-Principles Simulations

Ab initio molecular dynamics (AIMD) simulations were performed in the canonical *NVT* ensemble, neglecting thermal expansion effects and employing two different simulation cells containing 40 and 320 atoms with periodic boundary conditions applied along the three Cartesian directions. The temperature in the AIMD simulations was kept fluctuating around a set-point value by using Nose–Hoover thermostats.^72,73^ Newton’s equations of motion were integrated using the standard Verlet’s algorithm with a time step of 1.5×10^–3^ ps. Γ-point sampling for reciprocal-space integration was employed in the AIMD simulations, which spanned approximately over 100 ps. These calculations were performed with the semilocal PBEsol exchange–correlation functional.^65^

### Harmonic Phonon Calculations

The second-order interatomic force constant matrix for the three selected materials and resulting harmonic phonon spectrum were calculated with the finite-differences method as is implemented in the PhonoPy software.^21,22^ 2 × 2 × 2 and 4 × 4 × 4 supercells with a dense **k**-point grid of 4 × 4 × 4 and 2 × 2 × 2 for BZ sampling, respectively, were employed for the phonon calculations of targeted structures. These calculations were performed with the semilocal PBEsol exchange–correlation functional.^65^ The nonanalytical term for polar materials^74^ was taken into consideration through Gonze’s method^75^ using the Born effective charges and dielectric tensor.

### Anharmonic Phonon Calculations

The DynaPhopy software^76^ was used to calculate the anharmonic lattice dynamics (i.e., *T*-renormalized phonons) of the three selected materials from AIMD simulations. The supercells and simulation technical parameters described above were used in these calculations.

A normal-mode-decomposition technique^77^ was employed in which the atomic velocities **v**_*jl*_(*t*) (*j* and *l* represent particle and Cartesian direction indexes) generated during fixed-temperature AIMD simulation runs were expressed like

2 $$v_{jl}\left(t\right)=\frac{1}{\sqrt{Nm_{j}}}\sum_{qs}e_{j}\left(q,s\right)e^{iqR_{jl}^{0}}v_{qs}\left(t\right)$$

where *N* is the number of particles, *m*_*j*_ the mass of particle *j*, **e**_*j*_(q, *s*) a phonon mode eigenvector (q and *s* stand for the wave vector and phonon branch), R_*jl*_^0^ the equilibrium position of particle *j*, and *v*_q*s*_ the velocity of the corresponding phonon quasiparticle.

The Fourier transform of the autocorrelation function of *v*_q*s*_ was then calculated, yielding the power spectrum

3 $$G_{qs}\left(\omega \right)=2\int_{-\infty }^{\infty }\left\langle v_{qs}^{*}\left(0\right)v_{qs}\left(t\right)\right\rangle e^{i\omega t}dt$$

Finally, this power spectrum was approximated by a Lorentzian function of the form

4 $$G_{qs}\left(\omega \right)\approx \frac{\left\langle |v_{qs}{|}^{2}\right\rangle }{\frac{1}{2}\gamma_{qs}\pi \left[1+{\left(\frac{\omega -\omega_{qs}}{\frac{1}{2}\gamma_{qs}}\right)}^{2}\right]}$$

from which a *T*-renormalized quasiparticle phonon frequency, ω_q*s*_(*T*), was determined as the peak position, and the corresponding phonon line width, γ_q*s*_(*T*), as the full width at half-maximum. These calculations were performed with the semilocal PBEsol exchange–correlation functional.^65^ The nonanalytical term for polar materials^74^ was taken into consideration through Gonze’s method^75^ using the Born effective charges and dielectric tensor.

### Short-Wavelength Phonon Band Gap Correction

The electron–phonon correction to the band gap due to the short-range phonon modes was computed as the difference between the band gap at zero temperature for the static structure and the average band gap obtained from AIMD simulations performed with a supercell, namely

5 $$\Delta E_{g}^{S}\left(T\right)=\lim_{t_{0}\to \infty }\frac{1}{t_{0}}\int_{0}^{t_{0}}E_{g}^{R\left(t\right)}dt-E_{g}\left(0\right)$$

where **R** represents the positions of the atoms in the supercell at a given time *t* of the AIMD simulation. This expression can be numerically approximated as

6 $$\Delta E_{g}^{S}\left(T\right)=\frac{1}{N}\sum_{k=1}^{N}E_{g}\left(\{R_{k}\left(T\right)\}\right)-E_{g}\left(0\right)$$

where the band gap is averaged over a finite number, *N*, of configurations, as described in.^40^ Similarly, thermal effects on the dielectric tensor were computed.

These calculations were performed with the hybrid HSEsol exchange–correlation functional and considering spin–orbit coupling effects.^66,67^ Due to involved high computational expense, the total number of configurations used for the average was *N* = 10 for each material and temperature. These values were found to be appropriate for obtaining band gap results accurate to within 0.1 eV, as described in work.^9^

### Long-Wavelength Phonon Band Gap Correction

The electron–phonon correction to the band gap due to long-range phonon modes was computed using the Fröhlich equation for a three-dimensional polar material.^40,41,78,79^ This correction was determined as the difference in the shifts of the conduction and valence bands

7 $$\Delta E_{g}^{L}\left(T\right)=\Delta \epsilon_{CB}^{L}\left(T\right)-\Delta \epsilon_{VB}^{L}\left(T\right)$$

where ϵ_VB_ and ϵ_CB_ denote the valence and conduction band, respectively.

The shift of each band was computed using the Frölich correction

8 $$\Delta \epsilon_{i}^{L}\left(T\right)=\frac{2\alpha_{P}}{\pi }\hbar \omega_{LO}\tan^{-1}\left(\frac{q_{F}}{q_{LO,i}}\right)\left[2n_{T}+1\right]$$

where α_*P*_ represents the polaron constant, ω_LO_ the phonon frequency averaged over the three longitudinal optical Γ phonon modes,^80^ and *q*_F_ a truncation factor that can be approximated as Debye sphere radius. *q*_LO,i_ is defined as $\sqrt{2m^{*}\left(\omega_{LO}+\omega_{i}\right)/\hbar }$ , m* being the charge carrier effective mass and ℏωi the state energy. The term *n*_*T*_ is the Bose–Einstein occupation number corresponding to the average LO vibrational frequency, and the polaron constant can be computed as

9 $$\alpha_{P}=\frac{e^{2}}{4\pi \epsilon_{0}\hbar }\left(\frac{1}{\varepsilon_{\infty }}-\frac{1}{\varepsilon_{0}}\right){\left(\frac{m^{*}}{2\hbar \omega_{LO}}\right)}^{1/2}$$

where ε_*∞*_ is the high-frequency dielectric constant and ε_0_ the static permittivity of the system. The physical quantities entering this latter expression were determined with DFT methods. The electron and hole effective masses were computed using the parabolic approximation. For the LO phonon frequency, we used an effective value computed as the average of the three corresponding longitudinal modes; this simplification is well justified and consistent with other works.^80^ The dielectric constants were also computed with DFT methods and corrected to incorporate temperature effects (see works^9,40,41^ for additional technical details).

### Ab initio Tight-Binding Models

All-electron DFT calculations for ab initio tight binding models were performed with the WIEN2K software^81^ using the local-density approximation^68^ to the exchange correlation energy along with the linearized augmented plane wave method (FP-LAPW).^82,83^ The technical parameters for these calculations were a 10 × 10 × 10 **k**-point grid and a muffin-tin radius equal to *R*_MT_ = 7.0/*K*_max_, where *K*_max_ represents the plane-wave cutoff. Localized energy-resolved Wannier states^84^ were then obtained for the tight-binding calculations^85–87^ considering the relevant Hilbert space in the interval −10 ≤ *E* ≤ 20 eV around the Fermi energy.

## Supplementary Material





## Notes

materials-310-list.csv (CSV)
Temperature-dependent phonon population, electronic band structure obtained with different functionals, refined DFT calculations for candidate materials, list of all candidate materials and their main features (compressed file), tight-binding numerical values for selected electronic orbitals, electronic density of states for several candidate materials, estimated electric field for phonon distortion model, band gap comparison considering different DFT exchange–correlation functionals (PDF)
