# Supplementary material for: Band-Gap Tunability in Anharmonic Perovskite-Like Semiconductors Driven by Polar Electron–Phonon Coupling
Source: J Am Chem Soc. 2025 Sep 30;147(41):37506–20. doi: 10.1021/jacs.5c11968 (PMC12532205; doi:10.1021/jacs.5c11968)
Supplement: Supplementary file 2 [file ja5c11968_si_002.pdf]

Supplementary Information for "Band-Gap Tunability in Anharmonic Perovskite-like Semiconductors Driven by Polar Electron-Phonon Coupling"

Pol Benítez,[1,2] Ruoshi Jiang,[3] Siyu Chen,[3] Cibrán López,[1,2] Josep-Lluís Tamarit,[1,2] Edgardo Saucedo,[2,4] Bartomeu Monserrat,[3] and Claudio Cazorla[1,2]

[1]*Department of Physics, Universitat Politècnica de Catalunya, 08034 Barcelona, Spain*

[2]*Research Center in Multiscale Science and Engineering, Universitat Politècnica de Catalunya, 08019 Barcelona, Spain*

[3]*Department of Materials Science and Metallurgy, University of Cambridge, Cambridge CB3 0FS, UK*

[4]*Department of Electronic Engineering, Universitat Politècnica de Catalunya, 08034 Barcelona, Spain*

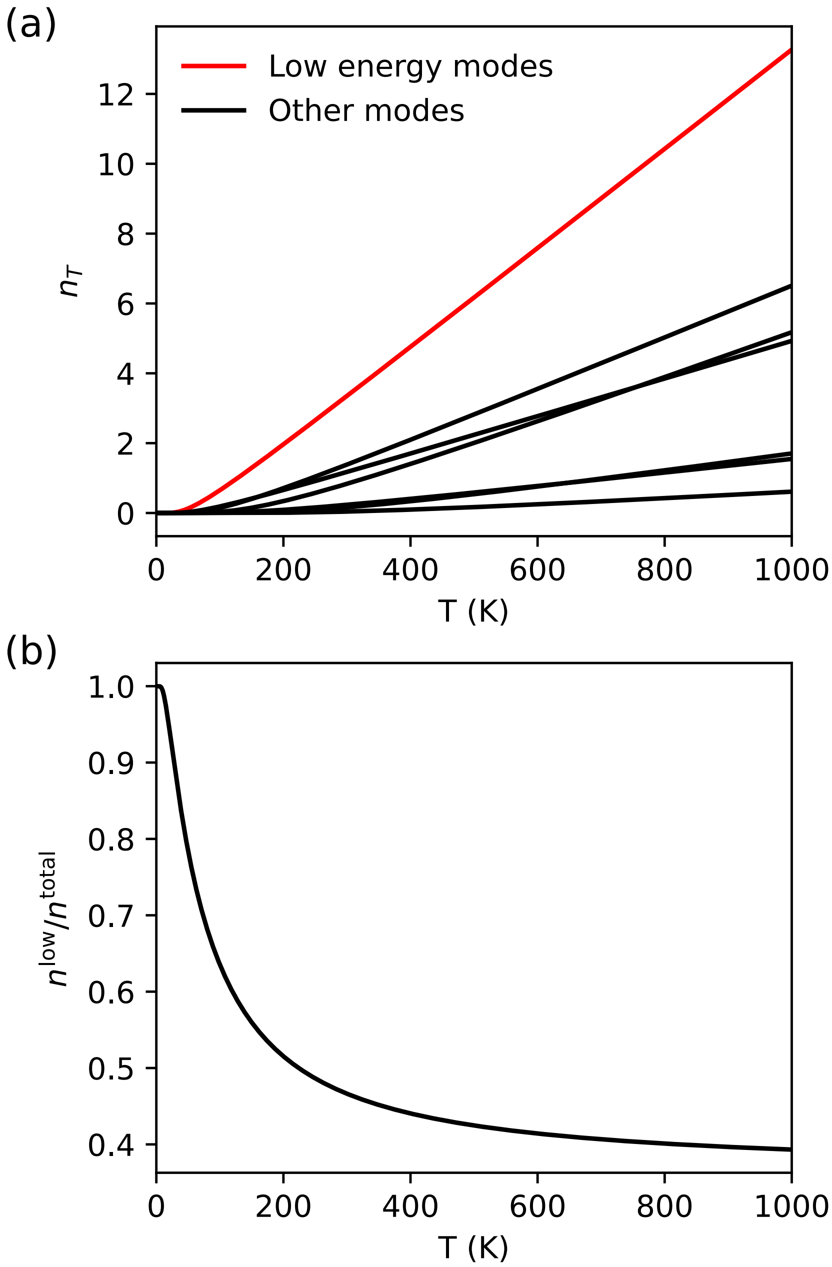

Supplementary Figure 1: (a) Bose-Einstein distribution for the different phonon modes at the Γ−point as a function of temperature. (b) Proportion of low-energy phonon modes relative to the total phonon population as a function of temperature.

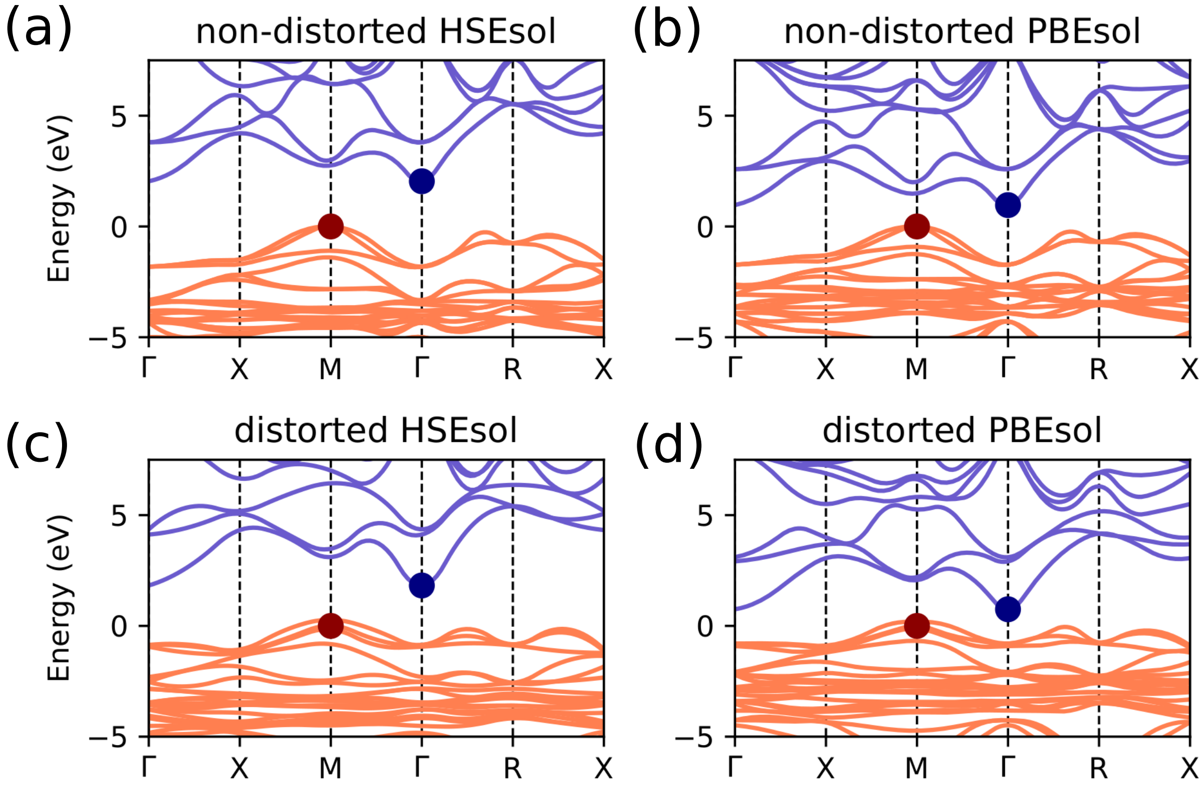

Supplementary Figure 2: Electronic band structures of the cubic $Pm\overline{3}m$ phase of Ag_3SBr computed using:(a) HSEsol and (b) PBEsol functionals for the undistorted structure, and(c) HSEsol and (d) PBEsol for the distorted structure ($u = 0.4$ Å). Orange lines represent valence bands and blue lines represent conduction bands. The red dot indicates the valence band maximum (VBM), and the blue dot indicates the conduction band minimum (CBM).

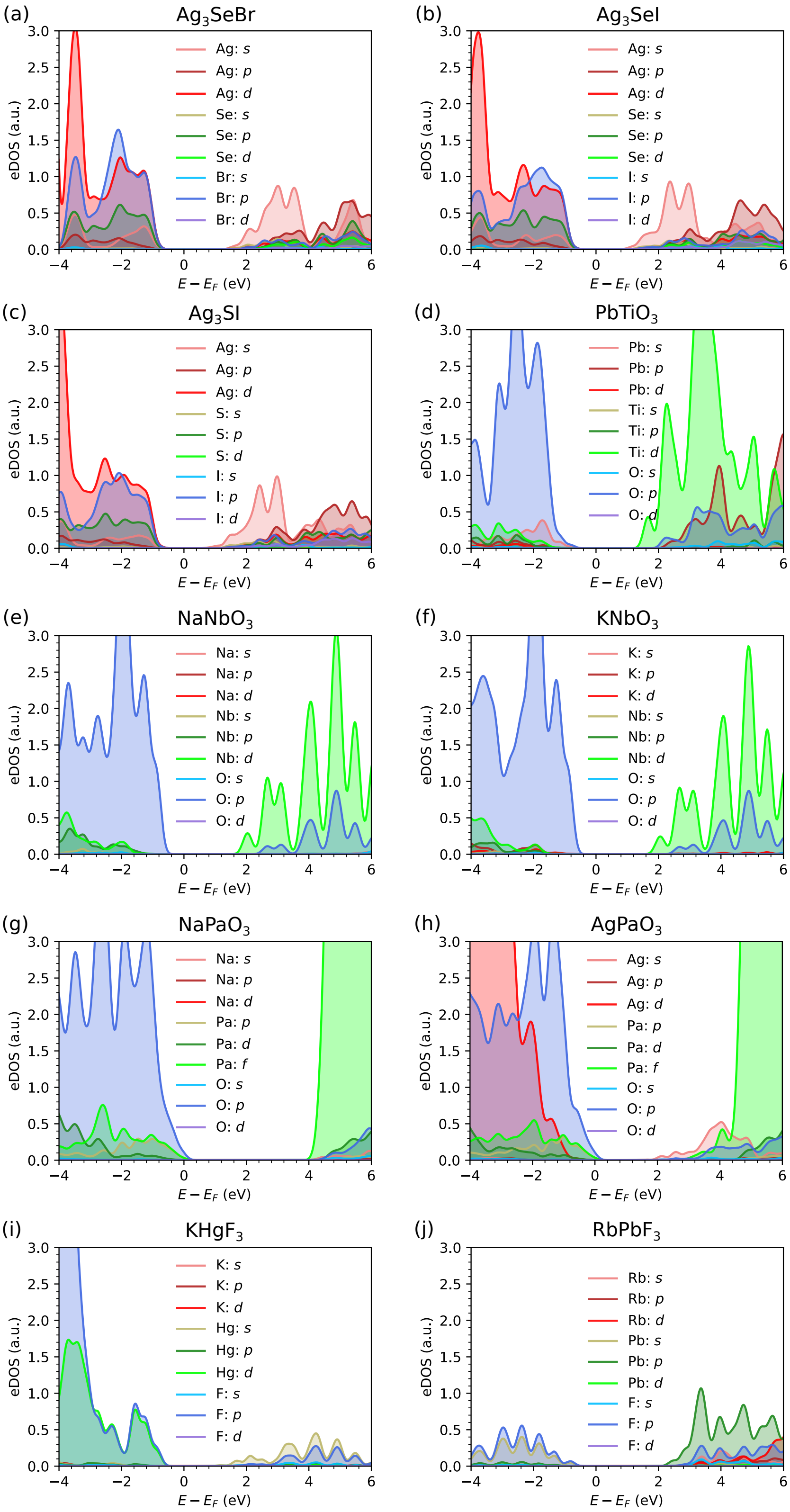

Supplementary Figure 3: Electronic density of states (eDOS) for the undistorted $Pm\overline{3}m$ structures of the following compounds (a) Ag_3SeBr, (b) Ag_3SeI, (c) Ag_3SI, (d) $PbTiO_3$, (e) $NaNbO_3$, (f) $KNbO_3$, (g) $NaPaO_3$, (h) $AgPaO_3$, (i) $KHgF_3$, and (j) $RbPbF_3$.

mp-id	Material	Space group	Imaginary phonon	E_{phonon} (meV)	Polar	E_{gap} (eV)
mp-2175	TlF	$Fm\bar{3}m$	Yes	12.745	No	2.57
mp-2998	$BaTiO_3$	$Pm\bar{3}m$	Yes	16.750	Yes	2.53
mp-3136	$NaNbO_3$	$Pm\bar{3}m$	Yes	21.355	Yes	2.51
mp-4419	$NaNbO_3$	$P4/mbm$	Yes	18.879	Yes	2.53
mp-5229	$SrTiO_3$	$Pm\bar{3}m$	No	-	-	2.66
mp-7483	$KHgF_3$	$Pm\bar{3}m$	Yes	8.387	Yes	1.79
mp-19845	$PbTiO_3$	$Pm\bar{3}m$	Yes	14.664	Yes	2.28
mp-21043	$RbPbF_3$	$Pm\bar{3}m$	Yes	10.410	Yes	3.57
mp-21276	PbS	$Fm\bar{3}m$	No	-	-	1.09
mp-22535	$PbHfO_3$	$Pm\bar{3}m$	Yes	15.089	Yes	3.19
mp-22977	$CsVCl_3$	$P6_3/mmc$	Yes	52.004	No	0.77
mp-22991	$CsVI_3$	$P6_3/mmc$	Yes	29.130	No	0.70
mp-23038	$CsVBr_3$	$P6_3/mmc$	Yes	39.635	No	0.94
mp-24341	$CsBeH_3$	$Pmmn$	Yes	29.931	No	3.68
mp-28650	$CsBr_2F$	$P4/mmm$	No	-	-	2.62
mp-37458	Na_2HF_4	$P2/m$	Yes	9.206	No	0.49
mp-626151	YHO_2	$P2_1/m$	Yes	23.169	Yes	5.54
mp-632711	$InHO_2$	$Pnnm$	Yes	20.355	Yes	3.18
mp-643043	RbHO	$P2_1/m$	Yes	41.949	Yes	4.69
mp-644225	Sr_2MgH_6	$P\bar{3}m1$	Yes	15.040	No	3.22
mp-865120	$NaPaO_3$	$Pm\bar{3}m$	Yes	11.237	Yes	4.46
mp-935811	$KNbO_3$	$Pm\bar{3}m$	Yes	24.427	Yes	2.37
mp-977455	$AgPaO_3$	$Pm\bar{3}m$	Yes	8.848	Yes	1.45
mp-981103	Sr_3CdO_4	$Pm\bar{3}m$	Yes	6.053	Yes	1.61

Supplementary Table I: Summary of properties for materials identified in our screening. Listed are the composition, space group after relaxation (PBEsol), Materials Project ID (mp-id), presence of imaginary phonon modes (including energy and polarity, if applicable), and HSEsol band gap. All materials are centrosymmetric and those that fulfill our screening criteria are underlined.

Material	Hamiltonian element	Non-distorted	Distorted
Ag_3SBr	$\langle \mathrm{Ag}_2\text{-}s \| H_{\mathrm{TB}} \| \mathrm{Ag}_2\text{-}s \rangle$	3.91 eV	4.35 eV
	$\langle \mathrm{S}\text{-}s \| H_{\mathrm{TB}} \| \mathrm{S}\text{-}s \rangle$	15.24 eV	14.05 eV
	$\langle \mathrm{Ag}_2\text{-}s \| H_{\mathrm{TB}} \| \mathrm{S}\text{-}s \rangle$	2.26 eV	2.39 eV
$BaTiO_3$	$\langle \mathrm{O}_2\text{-}p_y \| H_{\mathrm{TB}} \| \mathrm{O}_2\text{-}p_y \rangle$	-1.54 eV	-0.91 eV
	$\langle \mathrm{Ti}\text{-}d_{x^2-y^2} \| H_{\mathrm{TB}} \| \mathrm{Ti}\text{-}d_{x^2-y^2} \rangle$	4.08 eV	5.23 eV
	$\langle \mathrm{O}_2\text{-}p_y \| H_{\mathrm{TB}} \| \mathrm{Ti}\text{-}d_{x^2-y^2} \rangle$	-1.89 eV	3.22 eV
$PbHfO_3$	$\langle \mathrm{O}_3\text{-}p_x \| H_{\mathrm{TB}} \| \mathrm{O}_3\text{-}p_x \rangle$	-1.01 eV	-1.28 eV
	$\langle \mathrm{Hf}\text{-}d_{z^2} \| H_{\mathrm{TB}} \| \mathrm{Hf}\text{-}d_{z^2} \rangle$	9.46 eV	8.62 eV
	$\langle \mathrm{O}_3\text{-}p_x \| H_{\mathrm{TB}} \| \mathrm{Hf}\text{-}d_{z^2} \rangle$	0 eV	-0.75 eV

Supplementary Table II: Numerical values of the selected tight-binding Hamiltonian (H_{TB}) elements for the three studied compounds, Ag_3SBr, $BaTiO_3$, and $PbHfO_3$, before and after distortion.

	Electric Field (kV/cm)		
u (Å)	Ag_3SBr	$BaTiO_3$	$PbHfO_3$
0.2	0.07	0.39	1.46
0.4	0.15	0.79	2.92
0.6	0.22	1.18	4.38
0.8	0.30	1.58	5.84

Supplementary Table III: Electric fields required to excite a soft polar phonon mode to a targeted amplitude in three optoelectronic candidate materials.

Functional	Band gap (eV)
PBE	0.85
PBE+SOC	0.77
PBEsol	0.75
PBEsol+SOC	0.68
HSE06	1.96
HSE06+SOC	1.87
HSEsol	1.86
HSEsol+SOC	1.76
SCAN	1.20
SCAN+SOC	1.14

Supplementary Table IV: Theoretical band gaps calculated at $T = 0$ K conditions for Ag_3SBr using different DFT exchange-correlation functionals with and without considering spin-orbit coupling (SOC).

SUPPLEMENTARY DISCUSSION

Phonons, as bosonic quasi-particles, follow a Bose-Einstein distribution. The phonon population as a function of temperature is given by:

$$n(T) = \frac{1}{e^{\frac{E}{k_B T}} - 1}, \tag{1}$$

where E is the phonon energy, k_B is the Boltzmann constant, and T is the temperature.

From this distribution, it is clear that low-energy phonon modes will be more populated at a given temperature. Supplementary Figure 1a shows the Bose-Einstein distribution for all phonon modes at the Γ-point of $PbHfO_3$, taking into account their degeneracy. Low-energy optical phonon modes are highlighted in red, while the rest are shown in black. Due to their lower energy, the low-energy modes dominate the phonon population at lower temperatures. However, as temperature increases, their relative contribution decreases, as shown in Supplementary Figure 1b.

To compare the influence of hybrid versus non-hybrid DFT functionals on band-structure calculations, we computed the energy bands of Ag_3SBr in both the undistorted and distorted $Pm\overline{3}m$ phase using HSEsol and PBEsol. These are shown in Supplementary Figure 2. Although the band gap values differ, the overall band morphology remains consistent.

Supplementary Figure 3 shows the electronic density of states near the band gap for additional perovskite-like systems not explored in detail in the main text.

From our high-throughput screening, we selected the 24 candidate materials with 10 atoms or fewer in their unit cell for further analysis. For these, we refined the DFT parameters (ENCUT, KPOINTS) as specified in the Methods section of the main text. After structural relaxation, we computed their phonon modes at the Γ-point and evaluated their HSEsol band gaps.

Supplementary Table I presents the final results. For each material, we provide its Materials Project identity number, composition, relaxed space group, the presence of imaginary phonons at the Γ-point (if any), the corresponding phonon energy, and the HSEsol band gap. Materials that still met all screening criteria are underlined.

Supplementary Table II presents the numerical values of the Hamiltonians derived from tight-binding models for the three representative compounds, before and after distortion. The selected elements correspond to the on-site energy (diagonal) terms and the hopping (off-diagonal) terms for the most relevant orbitals near the valence and conduction bands. The general effect of structural distortion is an increase in the magnitude of the hopping terms and a reduction in the difference between the on-site energies of the two considered orbitals for each material. As discussed in the main text, this is related to an enhanced splitting between the bonding and antibonding states resulting from orbital hybridization.

To estimate the electric field required to induce soft polar phonon modes with varying amplitudes, we consider the potential energy within the harmonic approximation:

$$U(u) = \frac{1}{2}\omega^2 u^2 - Z^* u E \ , \tag{2}$$

where ω is the phonon frequency, u is the total displacement, Z^* is the Born charge, and E is the applied electric field. By imposing the minimum condition for the potential energy $\frac{dU}{du} = 0$, for a $u \neq 0$, the electric field corresponding to a given displacement can be expressed as:

$$E = \frac{\omega^2 u}{Z^*} \ . \tag{3}$$

The Born charge tensor can be reduced to an effective scalar value using the relation:

$$\mathbf{Z}^*_\beta = \sum_i \sum_\alpha Z^*_{i,\alpha\beta} \cdot e_{i,\alpha} \ , \tag{4}$$

where α and β denote Cartesian coordinates, i runs over the atoms in the unit cell, and $\mathbf{e}$ represents the normalized phonon eigenvectors. Supplementary Table III shows the electric fields required to induce one of the soft polar phonon modes considering various distortion amplitudes.
